# Quain Hernia Masquerading as Mesenteric Ischemia

**DOI:** 10.7759/cureus.61664

**Published:** 2024-06-04

**Authors:** Karthik N, Mahendra Lodha, Niladri Banerjee, Piyush Sharma, Priya Chhawal

**Affiliations:** 1 General Surgery, All India Institute of Medical Sciences, Jodhpur, IND

**Keywords:** bowel gangrene, mesenteric ischemia, laparotomy, broad ligament defects, quain hernia

## Abstract

The case describes a rare instance of Quain hernia, a specific type of internal hernia where the bowel protrudes through a defect in the broad ligament. Broad ligament defects can either be congenital or acquired. Quain hernias are uncommon and difficult to diagnose due to nonspecific symptoms. We report a case of a Quain hernia initially diagnosed as mesenteric ischemia with small bowel gangrene.

If a Quain hernia is suspected, immediate diagnostic laparoscopy is recommended, as it is an effective diagnostic tool and definitive management method, regardless of the specific type. During the laparoscopic procedure, it is crucial to thoroughly assess the contralateral broad ligament to identify any defects, which should be repaired prophylactically if found. Understanding their rare presentation and distinctive radiological features is vital for prompt diagnosis and appropriate management, highlighting the need to consider uncommon etiologies in acute abdominal cases to optimize patient outcomes.

## Introduction

Quain hernia is a specific type of internal hernia where the bowel protrudes through an opening in the broad ligament. Its recognition dates back to 1861 when an autopsy revealed the small bowel obstruction secondary to the broad ligament defect [1]. Broad ligament internal hernias are rare and often present with nonspecific symptoms. We report a case of a Quain hernia initially diagnosed as mesenteric ischemia with small bowel gangrene. This case emphasizes the diagnostic complexity of distinguishing uncommon etiologies during acute abdominal presentations and underscores the importance of considering rare etiologies in patients with atypical presentations.

## Case presentation

A 42-year-old multiparous lady with a medical history of hypertension, chronic kidney disease, and hypothyroidism presented to our emergency department with complaints of diffuse abdominal pain for a day, associated with multiple episodes of bilious vomiting. Recurrent episodes of colicky abdominal pain had previously led to hospitalizations. The patient has previously undergone two lower-segment cesarean sections. Physical examination revealed mild abdominal distension with diffuse tenderness and guarding. The patient had tachycardia, normal blood pressure, and 98% saturation on room air. Metabolic acidosis with a base deficit was noted on arterial blood gas analysis.

**Figure 1:**
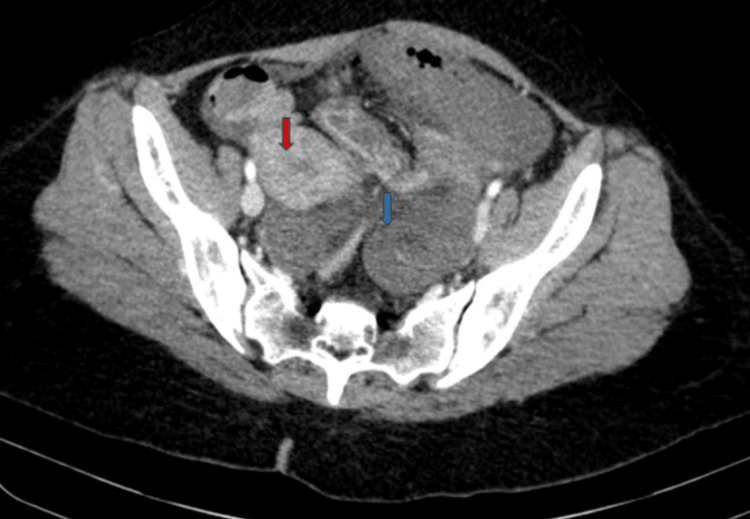
Computed tomography showing ischemic small bowel loops. In the axial plane computed tomography, there was evident diffuse circumferential thickening of distal ileal loops and suboptimal mucosal enhancement (blue arrow), all localized in the pelvic region. Red arrow showing anterior and right lateral displacement of the uterus.

**Figure 2:**
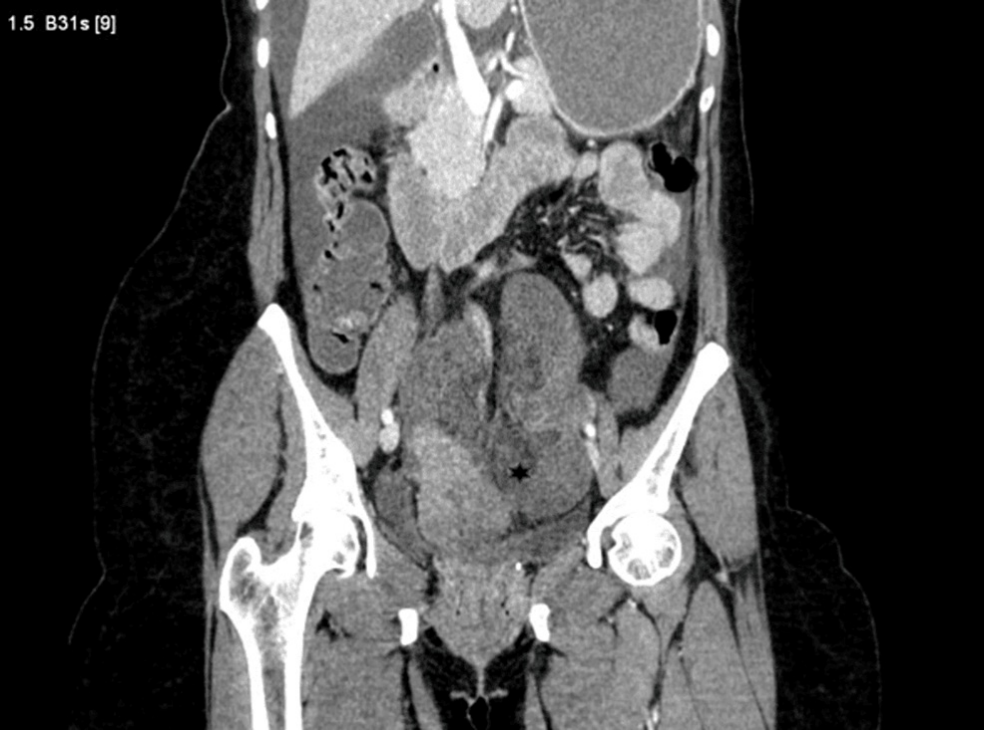
Computed tomography showing non-enhancing ileal loops. Coronal plane computed tomography demonstrating gangrenous changes of ileal loops within the pelvis (*) and mild dilatation of proximal small bowel loops. Additionally, a notable amount of moderate fluid was observed within the peritoneal cavity.

X-ray of the abdomen revealed dilated small bowel loops and significant air-fluid levels. Further, contrast-enhanced computed tomography of the abdomen with angiography suggested diffuse circumferential mural thickening of distal ileal loops in the left lower abdomen with poor mucosal enhancement (Figure 1). However, the reason for ischemic bowel loops was unclear. A small non-enhancing filling defect was seen in the infrarenal aorta without any significant luminal compromise, likely representing thromboembolic disease. However, the rest of the abdominal aorta, inferior vena cava, portal vessels, splenic vein, coeliac axis, superior mesenteric artery, and inferior mesenteric artery were well opacified with normal caliber. Additionally, moderate fluid was observed within the peritoneal cavity (Figure 2).

**Figure 3:**
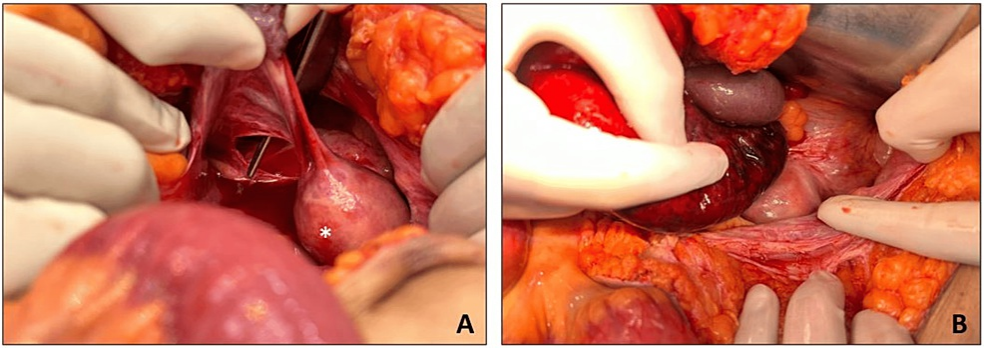
(A) Broad ligament defect. (B) Gangrenous ileal loops. A: A defect measuring 5 x 4 cm in the left broad ligament caudal to the round ligament (* denotes the uterus). B: Gangrenous distal ileal loops of length 100 cm herniating through the defect in the left broad ligament. The distal-most extent of the gangrene was the ileocecal junction.

Following resuscitation, the patient underwent exploratory laparotomy, which revealed a 5 x 4 cm defect in the broad ligament defect on the left side (Figure 3A). A 100-cm-long gangrenous distal ileal loop was found herniating through it, with the distal-most extent being the ileocecal junction (Figure 3B). The patient underwent resection of the gangrenous bowel, including the ileocecal junction, followed by a side-to-side isoperistaltic hand-sewn anastomosis of the ileum and ascending colon. The broad ligament defect on the left side was closed in a continuous fashion with polydioxanone 2-0, and no defect was found on the contralateral side. Anticoagulation therapy was initiated with unfractionated heparin infusion (18 units/kg/hour) for floating aortic thrombus. Due to bleeding episodes per rectum on postoperative day three, anticoagulation was temporarily halted. Oral dabigatran 150 mg twice daily was subsequently started, and the patient was discharged on postoperative day seven. The patient's recovery progressed uneventfully, and at the six-month follow-up, the patient remained stable.

## Discussion

Internal herniation resulting in small bowel obstruction is an infrequent occurrence, reported to have an incidence ranging from 0.2% to 0.9% [2]. Para duodenal hernias are the predominant cause of this condition in over 50% of cases [2]. On the other hand, Quain hernia constitutes only 4% to 7% of such instances [3]. Broad ligament defects can either be congenital or acquired. Acquired lacerations of the broad ligament can occur due to iatrogenic injury during cesarean section, rapid labor, instrumental deliveries, or trauma [4].

**Figure 4:**
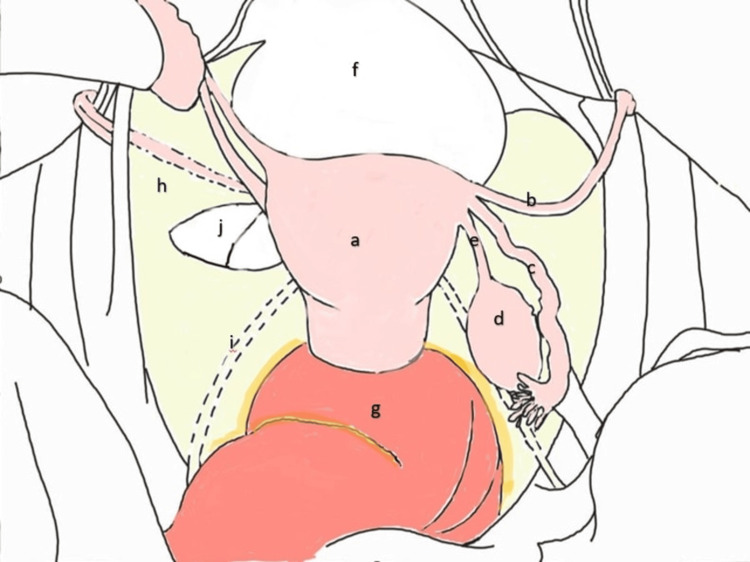
Anatomy of broad ligament. Superior view of the female pelvis. (a) Uterus; (b) round ligament; (c) fallopian tube; (d) ovary; (e) ligament of the ovary; (f) urinary bladder; (g) rectum; (h) broad ligament; (i) ureteric fold; (j) broad ligament defect caudal to the round ligament. Image credits: Karthik N.

There are two main classifications of broad ligament hernias. Hunt introduced a classification based on the degree or extent of the defect, including the fenestra and pouch types [5]. Our case was the fenestra type, with a complete two-layer broad ligament defect. Cilley classified it into three types based on the anatomical location of the defect [6]. According to Cilley’s classification, our case corresponds to a type 1 defect, specifically located caudal to the round ligament (Figure 4).

In our case, based on the cross-sectional imaging, the preoperative diagnosis was mesenteric ischemia with small bowel gangrene secondary to thromboembolic disease. However, adhesive obstruction with bowel gangrene, internal hernias, and small bowel volvulus should be considered in cases of bowel gangrene without evidence of any thromboembolic disease.

If an internal hernia is suspected, immediate diagnostic laparoscopy is recommended, as it is an effective diagnostic tool and definitive management method, regardless of the specific type. During the laparoscopic procedure, it is crucial to thoroughly assess the contralateral broad ligament to identify any defects, which should be repaired prophylactically if found. Indeed, increasing awareness regarding possible presenting symptoms and recognizing the distinctive radiological features associated with Quain hernia is imperative for minimizing morbidity and mortality.

## Conclusions

Broad ligament internal hernias, although rare, should be considered a differential diagnosis, especially in cases where initial findings suggest mesenteric ischemia without any evidence of thromboembolic disease. Appropriate early diagnosis of internal hernias with the help of cross-sectional imaging and timely minimal access surgery prevents complications and ensures a favorable outcome.
